# 

**Published:** 1975-04

**Authors:** 


					s
^...
.-. ?? ?'. ?.' ? .'?? ?.?.?; '??..? ?.'? -i- , ;
I HgUHKrara^gMl
- 'ft ?*5
ysfe^;: = V ?* zib?*M*
mm.
&wm
??a
ilS?ilii#a%iSI!
m
BRIS
. !_2r,
JOURNAL
mte
IBHSni
BWWWIiliiBiliWIIiiiWIIilliilWiWiilWWWBiBL^iWWBWiiliBillB...     _.1|-T.
?
NATIONi ??H
LIBRARY ^
?
m f: *w'
BkIMMS
!
^.'.s^stv "'.v*'". .>3
APRIL 1975
VOL. 90 (ii) No. 334

				

